# “We’re all going through it”: impact of an online group coaching program for medical trainees: a qualitative analysis

**DOI:** 10.1186/s12909-022-03729-5

**Published:** 2022-09-13

**Authors:** Adrienne Mann, Tyra Fainstad, Pari Shah, Nathalie Dieujuste, Kerri Thurmon, Kimiko Dunbar, Christine Jones

**Affiliations:** 1 Department of Medicine, Division of Hospital Medicine, University of Colorado Anschutz Medical Campus, Veterans Health Administration, Aurora, CO USA; 2 Rocky Mountain Regional VA Medical Center, 1700 N Wheeling St., Aurora, CO 80045 USA; 3 Department of Medicine, Division of General Internal Medicine, University of Colorado Anschutz Medical Campus, Aurora, CO USA; 4 Lowry Internal Medicine, 8101 E Lowry Blvd Ste 120, Denver, CO 80230 USA; 5 Graduate School of Social Work, University of Denver, Denver, CO USA; 6 Adult & Child Consortium for Health Outcomes Research & Delivery Science (ACCORDS), University of Colorado, School of Medicine, Aurora, CO USA; 7 Department of Surgery, Division of Urology, Denver Health, Denver, USA; 8 Department of Pediatrics, Division of Hospital Medicine, Children’s Hospital, Aurora, USA; 9 Veterans Health Administration, Eastern CO Health Care System, Denver-Seattle Center of Innovation for Veteran-Centered and Value Driven Care, Aurora, CO USA

**Keywords:** Coaching, Deliberate practice, Wellbeing, GME, Burnout

## Abstract

**Background:**

Trainees in graduate medical education are affected by burnout at disproportionate rates. Trainees experience tremendous growth in clinical skills and reasoning, however little time is dedicated to metacognition to process their experiences or deliberate identity formation to create individualized definitions of success and wellbeing. The purpose of this study was to understand the perspectives and experiences of trainees who participated in a 6-month, web-based, group coaching program for women residents in training.

**Methods:**

Better Together Physician Coaching is a six-month, self-paced, online, asynchronous, coaching program with multiple components including live coaching calls, unlimited written coaching, and self-study modules. Semi-structured interviews of seventeen participants of Better Together from twelve GME programs within a single institution in Colorado were conducted from May to June of 2021. All identified as women and had participated in a 6-month coaching program. Both inductive and deductive methods were used in collecting and analyzing the data with an aim to understand learners’ perceptions of the coaching program, including “how and why” the coaching program affected training experiences and wellbeing.

**Results:**

Three main themes emerged as benefits to the coaching program from the data: 1) practicing metacognition as a tool for healthy coping 2) building a sense of community, and 3) the value of a customizable experience.

**Conclusions:**

Female trainees who participated in a group coaching program expressed that they found value in learning how to cope with stressors through metacognition-focused coaching. They also described that building a community and being able to customize the experience were positive aspects of the program.

**Trial registration:**

ClinicalTrials.gov Identifier: NCT05280964. Date of registration: March 15th 2022. Retrospectively registered. URL of trial registry record.

**Supplementary Information:**

The online version contains supplementary material available at 10.1186/s12909-022-03729-5.

## Background

Burnout adversely affects physicians through higher rates of depression, suicidal ideation and substance abuse than the general population [1–3]. Physician burnout also adversely affects patients through higher medical errors and mortality rates [1, 2]. Burnout begins in medical school and worsens progressively over training, with female trainees being disproportionately affected [3–6]. Prior research in graduate medical education (GME) wellbeing has focused on the importance of creating a supportive learning environment [7]. However, despite efforts to improve learning and teaching methods, these strategies have not significantly mitigated burnout [8, 9].

The stress of the COVID-19 pandemic has added urgency to address this crisis [5]. Residency training is a critical time for development of personal and professional identity, but is limited by lack of dedicated time and space for emotional processing and metacognition (i.e., thinking about one’s thinking) which are key to identity development [10–12]. Furthermore, in an educational system that is primarily competency and outcomes-based, minimal attention is devoted to development of individual trainee values, visions and metrics of success, which are key factors in prevention and mitigation of burnout [13].

Various approaches to positively impact trainee wellbeing have been explored, including fostering reflective capacity through writing, and teaching mindful practices and resilient responses to challenging interactions [13]. One promising approach to improving GME wellness is professional coaching [4–18]. Coaching differs from mentoring, advising, and teaching in that it uses reflection and metacognition, rather than advice, to help the coachee manage their thoughts, feelings, and actions, to move toward fulfillment. Unlike therapy, coaching does not diagnose or clinically treat the coachee, and when supported institutionally, it is highly accessible [19].

Most previously described coaching interventions use variably trained coaches, and 1:1 in-person sessions which are logistically challenging and costly to incorporate and scale within GME programs [14–20]. A lesser explored alternative is group coaching [10, 11]. Group coaching models may offer opportunities to normalize mental health service utilization, share experiences, and build a sense of community, which are attributes that have been shown to positively affect trainee wellbeing [15, 21–23].

Building upon previously described evidence-based coaching interventions and metacognitive and adult-learning theory-informed coaching tools, two physician life coaches (AM and TF) created Better Together Physician Coaching (BT), a web-based, group-coaching program housed on a members-only website and piloted it among 101 female trainees during 2021 [24]. During the six-month program participants were taught and guided in the application of a metacognitive tool referred to as the CTFAR model (CTFAR) [25]. The CTFAR model offers a framework for participants to think about their thinking by separating and exploring the relationship between Circumstances, Thoughts, Feelings, Actions and Results. Participants in Better Together could access coaching through 1) twice weekly live group-coaching calls using the Zoom video-conferencing platform (Zoom Video Communications, Inc., San Jose, CA), 2) unlimited, anonymous written coaching in an online “Ask for Coaching” forum, and 3) weekly self-study modules (videos and accompanying worksheets) on a range of topics pertinent to the trainee experience (e.g., adopting a growth mindset, welcoming feedback, impostor syndrome, and perfectionism) (Additional file 1: Appendix A). The BT program has been described in detail previously, and a pilot randomized controlled trial of BT showed statistically significant improvements in emotional exhaustion, imposter syndrome, and self-compassion in the intervention group compared with controls [24].

Following the 6-month pilot RCT, our team conducted a qualitative study with BT participants to increase understanding of learners’ perceptions, including “how and why” the coaching program affected their experiences and wellbeing.

## Methods

### Participants

Female-identifying, GME trainees at the University of Colorado (CU), a tertiary care center with academic, county, Veterans Administration, and community healthcare facilities were eligible for inclusion in BT. We used purposive convenience sampling to recruit participants. All residents who completed BT were invited via email to participate in a one-hour interview with one of two interviewers (PS, ND) within 1 month after the BT intervention ended. All interviews were completed by July 27th, 2021. Participation was voluntary and compensated with a $75 gift card.

### Study design

#### Data collection

This qualitative study was designed prospectively to complement the quantitative analysis in the overarching sequential mixed methodology used to study the BT intervention. Our quantitative analysis was completed prior to our qualitative analysis [24]. We decided a priori to conduct a qualitative analysis to seek feedback on the program structure and implementation, to revise and improve future iterations of the program based on participant feedback, and to describe in more detail the quantitative results [24].

This was a qualitative descriptive study of resident physician participants in BT using a mixed deductive and inductive analysis (Additional file 2). The researchers had both planned domains they analyzed as well as unplanned domains they explored openly. Deductively, the researchers planned to summarize responses using the CTFAR model, using overarching codes of “thought model”, “result model”, and “model applications” to categorize the different ways the model was applied by the participants. Further, each strand of the CTFAR model was coded to its components, “circumstances”, “thoughts”, “feelings”, “actions”, and “results” which were applied to the transcripts to draw understanding of what common situations the participants applied this model to. Similarly, the researchers used a deductive approach to obtain feedback on the program modalities. This was done using the overarching codes of each of the modalities offered in the program, “live coaching”, “one on one coaching”, “getting coached”, “webinars”, “worksheets”, and “ask for coaching (written)”. These deductive frameworks lead to the themes of “Healthy Coping through Metacognition” and “Customizable Experience”. In turn, the inductive analytic approach led to the theme of “Building Community”, which emerged from several codes such as, but not limited to, “safe space”, “community”, “not alone”, “affirming”, “validating”, and “empowerment”. We developed a semi-structured interview guide to gather perspectives on reasons for joining, program expectations, application of the coaching model, program satisfaction, feedback on modalities, and general reflections (Additional file 1: Appendix B). The interviewers, both experienced with qualitative methods, piloted the interview guide. Consent was obtained from participants and interviews were conducted using Zoom conferencing platform, de-identified, professionally transcribed, and securely stored on CU’s REDCap platform. We collected data until thematic saturation was reached [26–28]. This study followed the Consolidated Criteria for Reporting Qualitative Research (COREQ – See Additional file 3) reporting guideline for trial studies and was reviewed and deemed exempt by the Colorado Multiple Institutional Review Board. This study was registered on ClinicalTrials.gov on 03/15/2022, Identifier: NCT05280964.

#### Data analysis

To assess emerging themes in real time and identify thematic saturation, a rapid domain analysis strategy was integrated into the qualitative analysis plan [29]. Interviewers completed a rapid domain analysis form after each interview. Both interviewers coded each interview independently. Consensus of common themes was reached through iterative discussions. Analysis was conducted using Atlas.ti Version 9.07 (Berlin, Germany). The researchers began deductive analysis and open-coding of the transcripts using the first 3 interviews to develop the codebook. Two coders (PS, ND) completed the entire coding process. Thematic decisions and content related questions were brought to the research team for discussion. We used elements of constructivist grounded theory to inductively code the data for elements outside of the coaching framework [30].

Transcripts of the interviews underwent constant comparison, iterative data reduction, and analysis by the coders, and occurred while interviews were ongoing, to allow for mutual influence. Coders coded line by line, consolidating initial codes to identify categories, and met regularly to review the resulting categories, corresponding analytical memos and overarching themes and their relationships to one another. After coding all 17 interviews, analysis continued beyond thematic saturation and purposefully explored counter examples in our data. We did not find any additional insights requiring changes to the coding structure, suggesting that our sample was sufficient for our study purpose.

Discrepancies were resolved through a process of deliberation until consensus was achieved. Our research team is made up of all female-identifying people of varying racial, age, socioeconomic, and career backgrounds. Our intersectional identities influenced how we made meaning of the data, enabling us to relate it to our own experiences and intentionally explore discrepancies in the data. Researcher memos were utilized to reflect on our own experiences in relation to collecting and analyzing the data.

The Consolidated Criteria for Reporting Qualitative Studies (COREQ) was used to report our findings (Additional file 3) [31].

#### Trustworthiness and credibility

These themes were brought to the entire research team to agree upon before completing analysis. Member checking was completed to ensure the results accurately reflected participant experience.

## Results

**Fig. 1:**
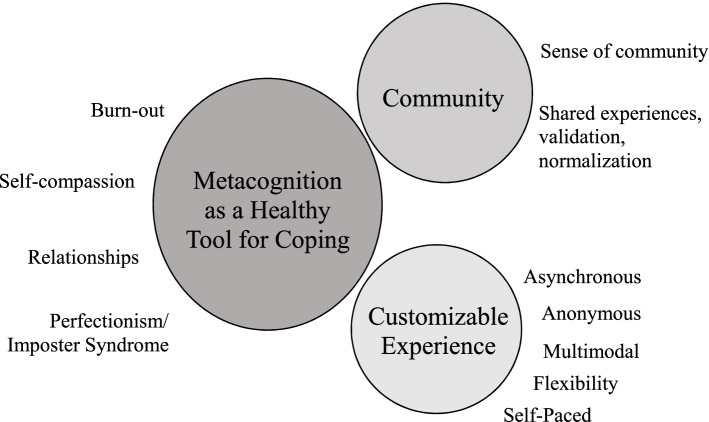
Impact of coaching on physician wellbeing

Of the 50 BT participants, 22 responded to interview invitations, and 17 completed interviews (Table 1). The participants ranged in age from 25 to 32, with an average post-graduate year of 2.35; the participants were majority white, and all were cis-female, and heterosexual. Seven different specialties were represented: otolaryngology, family medicine, general surgery, internal medicine, neurology, obstetrics and gynecology, and psychiatry. No participants who volunteered were excluded from the study. The interviews ranged from 26 to 70 minutes, with the majority lasting for 1 h. Three major themes about Better Together emerged from the interviews: 1) healthy coping through application of the CTFAR model as a tool for metacognition; 2) community building; and 3) accessible, customizable program design (Fig. 1). Within the major theme of metacognition through the CTFAR model, we identified four sub-themes related to burnout, growing self-compassion, managing impostor syndrome and perfectionism, and improving relationships. See Table 2 for exemplary quotes.

**Table 1:** Participant demographics

Characteristics of Interview Participants	
Participants (***N*** = 17)	
Age (m, range)	28.7 years old (25–32)
Post-Graduate Year (m)	2.35 years
PGY 1 (n, %)	3 (17.6%)
PGY 2 (n, %)	7 (41.2%)
PGY 3 (n, %)	5 (29.4%)
PGY 4 (n, %)	2 (11.8%)
Gender Identity	
Cis-female (n, %)	17 (100%)
Sexual Orientation	
Heterosexual (n, %)	17 (100%)
Racial/Ethnic Identity	
White (n, %)	12 (70.6%)
Asian (n, %)	4 (23.5%)
Prefer Not to Say (n, %)	1 (5.9%)
Residency Specialty	
Ear, Nose, & Throat (n, %)	1 (5.9%)
Family Medicine (n, %)	2 (11.8%)
General Surgery (n, %)	3 (17.6%)
Internal Medicine (n, %)	6 (35.5%)
Ob/Gyn (n, %)	2 (11.8%)
Neurology (n, %)	1 (5.9%)
Psychiatry (n, %)	2 (11.8%)

**Table 2:** Exemplary quotes that contribute to participant experiences of better together

Theme	Quote
Usefulness of the Metacognitive Coaching Model	
Subtheme: Burnout	"Wow, I really do resonate with [this coaching] about not feeling good at work and feeling frustrated with my patients and feeling these feelings that I've never felt before. I was like, I really do feel burnt out." (Participant 3) "Being able to separate my circumstance from my thoughts made me feel like there was hope, and my thoughts are just thoughts, and I can work on changing them and that would give me a different result and ultimately end in me trusting myself a little bit more." (Participant 4)
Subtheme: Self-Compassion	“I got something out of the program, [and] it was to be a lot more gentle with myself” (Participant 3). “I think having that grace towards yourself definitely can make you more confident as a physician … more happy as a physician. I definitely love what I do, but that doesn’t mean there’s not challenges … having these frameworks helps with being more equipped when there’s something tough that comes up” (Participant 4). “[BT] has also just helped me tell myself it’s okay to have a personal life again. I think when you get into some of these cycles, you think like, “Oh, I’m not working hard enough,” or, “I’m not good enough, and so I need to be doing all these other things outside of work.” I just had a lot more confidence to say like, “Nope. Today’s my day off. I deserve this.’” (Participant 3) “Every single day something brings me a feeling of joy, being like, I love being here. I get to like deliver babies, so I get to bring people’s greatest joy into the world. But second year with the pandemic and just ... there was like lots of like bad pregnancy outcomes and things like that. But [by the end of BT] I was like, wow, I do feel like I’m sort of moving towards not feeling so bad every day. Not every day feels just like quite as heavy as it did before.” (Participant 15)
Subtheme: Imposter Syndrome and Perfectionism	“[Prior to BT] I thought that constructive feedback meant that I was a bad doctor, and that I was bad at operating, whereas she was literally just helping me. And it was funny that I finally realized that my thoughts were, oh, I should be perfect at everything. I should know everything and no one should ever give me any feedback, like this unrealistic expectation that I didn’t even realize.” (Participant 6). “[I used to think] ‘Oh, I made the wrong decision, therefore I am a bad doctor’ or, ‘I did the wrong diagnosis or triaged this person to the wrong place’ and just heavily internalized that and felt worse. I feel so much more strongly now that I allowed myself to not be perfect on those shifts, and like what I was just saying before, it just was a growing period and it helped me when I left the hospital leave that tough shift behind me.” (Participant 11) I think I just allowed myself to be imperfect sometimes, and now I feel like I don’t even like saying “imperfect.” I don’t like focusing on the things I may have thought as bad before at all. I think I’ve really shifted my mindset to say anything that maybe had a poor outcome or wasn’t what I wanted is all just a growth period for me. (Participant 12) I had stated my thoughts and feelings around doing procedures was like, “I’m not going to be good at it,” … And from the coaching session, their big thing was taking those thoughts and feelings that I have about myself and then separating them from actually the circumstance ...So, instead of thinking, “I am not good at this procedure because I don’t know how to do it,” changing it to, “I am learning to do this procedure.” (Participant 3)
Subtheme: Improving Relationships	“I’ve made some improvements with shifting slightly from my avoidance [in relationships] to being more proactive. I am not fully satisfied with where that’s at, but I definitely think it nudged me in some way. … [For example] there could be a million different reasons why he is acting this way. And maybe also he’s just a human being and wants to be quiet for a little bit. Rather than jumping to conclusions or some crazy story about it, if I’m not sure, then I can ask him what’s going on …. And then often I would get the answer and it was never about me.” (Participant 11) “I had two male seniors who would make jokes or make comments or demand more of me. And I felt like I was working really hard but still like not being respected... so we worked through a different model, for a different result...and I wrote in my planner, like their thoughts about you are their models and what they have going on and their thoughts about you are actually no reflection of how you are as a doctor or a teammate, or any of that. So really being able to separate these other people from my life and not letting them have as much control over my thoughts or any control over my thoughts was so helpful.” (Participant 6)
Community	“I think connection to people, feeling … a sense of community, or that we all share something that we’re going through. Learning based on their experiences.” (Participant 8) “I don’t know if the program would have been different if it wasn’t for COVID. Instead of Zoom, these groups would be in person … that would have been a good idea because it’s nice to [feel] human interaction and bonding … the nice thing about the Zoom was that when you went up there [for coaching], you didn’t see anyone else looking at you. You kind of felt you were privately talking to someone.” (Participant 9) “I think a huge strength is just being able to see, ‘... although some things in my experience are unique, a lot of it isn’t’... [It’s] something that we all share being residents, working really hard, feeling inadequate at times, wanting to be perfect, feeling like we are going to get found out … it was helpful to look around and be like, ‘Oh, there’s all these amazing women who are awesome doctors who also feel all the same things …’” (Participant 8) “A huge benefit of it was having so many residents in different specialties, because you get so caught up in your world of surgery, and you think that you’re the only residents that work hard and feel miserable sometimes. So just hearing people and lots of other programs talking about very, very similar issues just felt super validating and made me feel like I was connected to a bigger pool of trainees.” (Participant 13)
Customizable Experience	
Sub-theme: Multimodal	“...allowing multiple ways to interact with the program [was helpful]. So not only the live coaching, but the ask-for-coaching and then being able to watch the videos on your own time, and then some people participated by just watching others. I was someone who was pretty regularly being coached live, which I found nerve wracking at first, but then probably the most beneficial part” (Participant 10). “… it’s nice being able to have a format that you can ask [for coaching] at any time and then also having a written response so that you can go back to it if you need to.” (Participant 8)
Sub-theme: Self-paced	“Access and availability...if I was in an inpatient rotation where I just was not free by the time [the call] started, I could go back and listen to it later. I read through some of people’s coaching requests. I was like, ‘Oh, yeah. This is useful for me, too’ in addition to other curriculum that you can go at your own pace for” (Participant 4) “I think that it was nice that you could pick and choose from the level of commitment that you wanted.” (Participant 5) “It was nice that you could pick and choose from the level of commitment that you wanted.” (Participant 2)
Sub-theme: Option for Anonymity	“...[live coaching] wasn’t a good fit because a lot of my co-residents were around. But I still feel like I benefited from watching other people get coached” (Participant 12). “So I really liked that they had an anonymous ask for coaching thing. I thought that was great.” (Participant 8)

### Healthy coping through metacognition

The participants shared numerous ways in which BT allowed them to practice metacognition as a healthy coping mechanism both within and outside of the coaching sessions. Specifically, residents reported that the CTFAR model provided a tangible and accessible tool to apply to their daily lives, directly pertaining to the sub-themes of burnout, self-compassion, imposter syndrome, and managing interpersonal relationships.

#### Burnout

Many trainees said that they were drawn to BT because of burnout, especially during the global COVID-19 pandemic. They shared that the program countered burnout by mitigating emotions of anxiety, frustration, feeling overwhelmed and worry. By processing and then letting go of these emotions, they were able to reduce the overall feeling of burnout in their work:

*“I was in just a lot of really busy rotations. A lot of really, really sick people all of January, February, and March, and at the end of March was just feeling very, very burnt out. I think a lot of the tools did help me to say, like when I finally had a day off, I wasn’t making myself work on research on those days or necessarily make more teaching plans or something that I may have done on a day off before. I think I had a lot more self-compassion to say like, “No, you have been working really hard and it’s okay to recognize that. You should take today off” (Participant 5).*.

#### Growing self-compassion

The program created space to practice self-compassion. Some trainees shared:

*“...that grace that’s granted when you ask yourself … “What would be enough?“ I think just hearing that phrase, “what would be enough for you to say that you’ve done enough for today or to have met this goal and realizing that it didn’t have to be 100% but to still be acceptable to myself” (Participant 4).*

#### Managing imposter syndrome and perfectionism

Throughout the interviews, trainees reported experiences of imposter syndrome in tandem with the experience of perfectionism. Through BT, trainees learned to re-evaluate their expectations for themselves. Many trainees shared that this reframing allowed them to remember that they are growing and learning, as in the following quote:

*“[Because of the program] I just allowed myself to be imperfect sometimes, and now I … don’t even like saying ‘imperfect’. I don’t like focusing on the things I may have thought were bad before at all … I’ve really shifted my mindset to say anything that maybe had a poor outcome or wasn’t what I wanted is all just a growth period for me” (Participant 5).*

#### Improving relationships

Relationships in and out of the workplace impacted trainee wellbeing. Trainees shared that challenging work relationships made it harder to learn, make decisions, or have difficult conversations. Participants reported that applying what they learned in the coaching program enabled them to improve both professional and personal relationships:

*“It helped with my overall life in regards to my relationship with my boyfriend and … with my parents..” (Participant 11).*

### Building community

Inductive findings from the interviews revealed the overall theme of community building which included the sub-themes of a sense of community and shared experiences with other participants. While the BT coaching program caters to individual preferences and needs, participants felt that the program built rapport, trust, and belonging. Hearing each other’s experiences offered a sense of validation and normalization that participants had not previously experienced. The trainees described feeling less isolated through these shared experiences. Whether they attended the calls or not, they felt the connection with their peers that gathered virtually and shared a safe space together:

*“Other people can understand me. [They] were able to vocalize a lot of things that I was thinking” (Participant 9).*

*“We all kind of came together to support each other. You’re not alone. So I think that was one of the huge strengths was that we could comment for each other and support each other too.” (Participant 11).*

### Customizable experience

BT was created for trainees to be customizable, accessible, and user-friendly. Participants appreciated this and frequently described that it met their individual needs and expectations. Specifically, they valued: the multiple modalities in which they could engage with the program, the ability to self-pace the coaching program materials, and the option to have anonymity.

#### Multimodal

The most common aspect of the program that participants enjoyed was the multiple modalities available for coaching. These options made the experience customizable to individual schedules, needs, personalities, and learning styles. Some shared:

*“...It’s nice being able to have a format that you can ask it at any time and then also having a written response so that you can go back to it if you need to” (Participant 8).*

#### Self-paced program

The participants commented that they appreciated the self-paced, asynchronous, flexible, and anonymous attributes of the program. When discussing time commitment for the program, the participants felt that BT allowed them to engage in which parts made the most sense for them. Many agreed that they could not manage engaging with all modalities simultaneously, but that engaging with those that fit their independent needs was impactful. One participant shared this about the self-study webinars and worksheets:

*“I thought they were very brief and to the point, so I could break up stuff into small chunks and I would learn about it. And then I can take some time to either reflect about what they were talking about or just go right to the worksheet” (Participant 7).*

#### Option for anonymity

Finally, the option of anonymity was appreciated in the live coaching and written ask-for-coaching modalities. While the live coaching calls were not anonymous for the person being coached, the other participants on the call could remain anonymous.

*“So, I really liked that they had an anonymous Ask-for-Coaching [forum], or an ability to set up just a one-time meeting with them on their own. I thought that was great” (Participant 9).*

## Discussion

In this qualitative study, we describe perspectives of participants in a novel web-based, group coaching program that was developed to promote trainee wellness. We found that providing a tool for metacognition (the CTFAR model) supported wellbeing. In addition, participants described a sense of community not otherwise experienced in training. Finally, participants appreciated the multiple modalities for coaching that allowed for a customizable experience accommodating learners with variable schedules and learning preferences.

Professional coaching is relatively new in academic medicine and has been shown to improve burnout and wellbeing among physicians and trainees [14, 16, 17, 19, 20]. Much of the existing coaching literature describes individual (1:1) coaching sessions and focuses on quantitative measures of wellbeing outcomes rather than understanding the facets that may contribute to their success [14–17, 20]. Our findings add to the literature by identifying concrete mechanisms by which this coaching intervention supported wellbeing among participants.

First, participants reported that learning and applying the CTFAR model for metacognition (thinking about one’s thinking) allowed them to gain insight, choice, and control over their training experience. These findings are consistent with available literature on burnout mitigation [32, 33], and the positive impact of guided inquiry around perceptions, beliefs, and habits to help the coachee move towards individually defined goals [14–17]. Others have described pedagogic strategies including interactive fostering of self-reflection and emotional awareness as tools to support professional identity formation in medical education [13]. Both of these strategies were incorporated into the metacognitive CTFAR model used in BT. Participants described that application of this model led to meaningful reflections on self-compassion, impostor syndrome, perfectionism, and relationships. Through the program, they described experiencing improvement in their interpersonal relationships with patients, peers and supervisors with deliberate alignment with their personal and professional values.

This study also provides insight into the potential role for group-coaching as a wellbeing intervention for physician trainees. Group coaching has been shown to support professional identity formation, and management of healthy work/life balance for junior physicians [11]. Our study affirms these findings and also describes how group-coaching fosters community and normalizes the challenges of residency training even in a web-based setting. Participants reported that by watching each other receive coaching, they felt less alone in challenges that were usually veiled in inadequacy or shame.

Finally, participants reported that the multi-modal and asynchronous structure of BT was a major benefit. Our modalities included live group coaching, anonymous written coaching, and self-study modules, which has not previously been described in the literature. Participants described engaging with BT when they had the availability and interest, which is unique compared to other wellness interventions which take place at a specific time and place, require 1:1 coordination, and may or may not be convenient for a resident schedule. Participants could engage as little or as much in any of the three modalities as they wished, allowing them to use BT based on their individual learning styles, schedules, and preferences. Resident trainees have little agency or autonomy over their schedules and day-to-day workload, and BT offered both brief on-the-spot reflection (using the website resources on-demand) or deeper self-inquiry (by attending or watching recorded coaching calls) depending on the time and needs of the trainee.

Some of the facets of BT such as deliberate mindfulness and reflection and fostering of self-compassion have been described as best practices in wellbeing interventions for physicians [34, 35]. Our study fits these well-known practices into a greater framework by describing *why* these program characteristics work: highlighting the experience of the trainees and demonstrating how coaching tools are useful in their personal and professional lives. Our study adds to the growing body of evidence recognizing coaching as an important tool for promotion of physician wellbeing. Our findings also suggest that the group coaching format was a positive factor which may help to normalize experiences and create community while also mitigating logistical challenges of 1:1 coaching interventions.

### Limitations

This was a single institution study of a coaching intervention for GME trainees who identify as female. Inclusion of women in this program was intentional, as this population is more impacted by burnout. The participants were representative of the demographics of female GME trainees at our institution, which are majority white, heterosexual, and cis-gender. As a result, these findings are not generalizable to trainees at other institutions that differ from our sample.

Both AM and TF are internal medicine physicians in the institution in which this study was completed. It is possible that their positions impacted recruitment of participants through a social desirability bias which may have encouraged trainees to volunteer (or not) for this study.

Finally, the changing nature of the COVID-19 pandemic may affect the context in which our data can be applied. Also, it is important to note the barriers to participating in BT: some participants reported busy trainee schedules (i.e., working night shifts), as an obstacle. Others noted feelings of isolation or lack of accountability in this self-paced program and reported a preference of more structure. Further, some participants shared feedback for the program such as, use of a different technology platform, tagging online material with keywords, having more worksheet examples, and having a structured coaching sign up. It is possible that the additional stress and social isolation for trainees created by the pandemic amplified both the need for and the effect of the coaching program.

## Conclusion

A physician-led group coaching program teaching metacognition enabled participants to reflect on and reframe beliefs around their work and professional lives to promote wellbeing. The community created by the program allowed for processing of burnout, growth of self-compassion, development of skills to address impostor syndrome and perfectionism, and improvement in relationships, all in a community that normalized the challenges. Having a customizable experience with multiple modalities for participation allowed participants with complex and irregular schedules and varying learning and communication preferences to benefit from the program. Group coaching may offer a meaningful and highly scalable strategy to mitigate burnout, foster community, and provide an accessible and customizable experience for trainee physicians.

## Supplementary Information

**Additional file 1.** Appendix.

**Additional file 2. **Study Protocol.

**Additional file 3. **Consolidated criteria for reporting qualitative studies (COREQ): 32-item checklist.

## Data Availability

The datasets” used and/or analyzed during the current study are not publicly available due to the ethical and privacy reasons around the sensitive nature of the material but are available from the corresponding author on reasonable request.
