# Supplementary material for: “We’re all going through it”: impact of an online group coaching program for medical trainees: a qualitative analysis
Source: BMC Med Educ. 2022 Sep 13;22:675. doi: 10.1186/s12909-022-03729-5 (PMC9468533; doi:10.1186/s12909-022-03729-5)
Supplement: Supplementary file 1 — Additional file 1. Appendix. [file 12909_2022_3729_MOESM1_ESM.docx]

**Appendix A.**

*Program Modalities*

*Live Coaching Calls.* Live coaching calls were facilitated by two certified coaches on the Better Together team. The coaches were certified through The Life Coach School^TM^, an institution for thought-based coaching, focused on metacognition and beliefs. Calls were hosted on the Zoom teleconferencing platform, using the “webinar” format, and were 60-minutes each. The “webinar” format of Zoom allowed participants to not know who the other participants were on each call unless someone got coached. There were two live coaching calls hosted each week, both on weeknights after work. During each live coaching call, participants could “raise their hand” to indicate a request to be coached while the other participants on the call could observe. Between 1-5 participants were coached per call, and participants were not required to raise their hands or ask for coaching unless they wished to. Participants were also able to engage in the coaching session via the Zoom chat feature. Questions or comments submitted in the chat were visible to all attendees. The coaching calls took place twice a week, and each coach led one session a week. The call recordings were saved in a secure folder accessible only to Better Together participants so they could engage with the live coaching calls when they could not attend them.

*“Ask-for-Coaching” Written Coaching.* The secure website houses an online forum where participants could request coaching via a written, anonymous format. Responses to written coaching requests were posted to the forum within 1-business day by a Better Together coach. This asynchronous format allowed access to coaching despite the ever-changing and round-the-clock schedules of trainees and provided a kinesthetic learning format for participants to engage with the coaching material. Ask-for-Coaching could be accessed at any point throughout the 6-month intervention, and was completely anonymous; the submitting participant was not identifiable to other participants.

*Worksheets.* Each month a new Better Together module opened with weekly worksheets that laid out each week’s theme (i.e., receiving feedback, setting goals, adopting a growth mindset, or defining work-life balance). These were provided in both a digital and a hardcopy format. Hardcopies were mailed to participants. The worksheets walked the participants through metacognitive techniques of awareness and processing feelings. The worksheets were also voluntary and meant for independent self-study.

*Webinars.* Short 5–10-minute videos corresponding to weekly content were released at the start of each month. The webinars were chalk-talk style didactics given by the Better Together coaches to introduce our weekly themes. The webinars served as an asynchronous component of the program in which participants could independently receive information.

**Appendix B.**

*Interview Guide*

**Better Together Qualitative Interview Guide**

*Introduction: I am [Pari Shah or Nathalie Dieujuste], and I have been asked by Dr. Adrienne Mann and Dr. Tyra Fainstad to conduct interviews with the Better Together Positive Psychology Coaching Program participants. We hope to understand ways in which the coaching program was beneficial and ways in which the program can be improved for the future. The Better Together Coaching Program hopes to implement tangible and meaningful improvements to the program based on this feedback.*

*I would like to ask you some questions about your experience with the Better Together Coaching Program. A transcript of your anonymous responses will be reviewed, per your consent, by me, Dr. Mann and Dr. Fainstad and research assistants, to create a summary of findings. Under no circumstances will your identity or the transcript of your responses be revealed. Your identity will be kept* ***confidential****, except in the circumstances we identified during the consent process, in which case, these concerns will be brought to the campus ombudsmen.*

*As previously mentioned, this interview will be recorded for transcription and analysis purposes.* ***Is this okay with you?***

*(Await participant consent).*

*Thank you. I believe our discussion will take about 45 minutes to 60 minutes, are you ready to begin?*

*(Await participant consent to begin the interview).*

****START RECORDER****

**Interviewer:** Today is [STATE DATE] and this is a qualitative interview for the Better Together Coaching Program.

*Before I begin, I want to clarify that when I ask you about the BT Coaching Program, I am referring to the entirety of the program (the live coaching calls, website, worksheets, ask-for-coaching written coaching, and YouTube webinars). You can answer for all OR any parts of that that you used. I will later have specific questions about each of those parts of the program.*

**Overarching Coaching Intervention Questions**

1. Why did you decide to enroll in the Better Together Coaching Program?

(Probe) What expectations did you have for the program?

1. Was there a specific moment when you knew the program was a good fit for you? Can you tell me about it?

(Probe) Was there ever a time when you realized this program was not a good fit for you?

1. What would you say were the strengths or highlights of the Better Together Coaching Program?

(Probe) Can you provide any examples?

1. What would you say were weaknesses or points of improvement for the Better Together Coaching Program?

(Probe) Can you provide any examples?

**Life Coach Framework & Construct Related Questions**

*Now I’m going to ask some questions about the specific type of coaching framework used in BT - feel free to skip any questions if you aren’t familiar with the terminology.*

**Separating Facts from Story, or the Circumstance from the Thoughts**

1. Tell me about a time, at work or at home, that you noticed your thoughts as separate from the circumstance? Sometimes we call this “separating the facts from the story”. It could have been something you got coaching on or not.

(Probe) How did this experience impact you?

(Probe) Did you decide to continue thinking that thought(s)?

**CTFAR Model: Circumstances, Thoughts, Feelings, Action, Results**

1. Tell me about a time, at work or at home, that you applied the “Thought Model (CTFAR)” to help work through an issue?

(Probe) How did this experience impact you?

(Probe) Did you use the model to bring awareness to your thoughts/feelings/actions/results? Tell me more...

**Result or Intention Model**

1. Tell me about a time, at work or at home, that you used a “result model” or “intentional model” to get a result that served you.

(Probe) Did you use the model to **create new** thoughts/feelings/actions/results? (Sometimes we call this a “result model”) Which one(s)? Tell me more….

(Probe) How did you find a thought that was believable that led to a better f/a/r? Can you share the thought(s)?

(Probe) Did you use the model to help create bridging thoughts to move into a different [intentional] model? Tell me more...

**Accept and Process Any Feeling**

1. Tell me about a time, at work or at home, that you were able to intentionally accept and process a feeling.

(Probe) What was the feeling? How did this experience go for you? Was the feeling uncomfortable? Was it tolerable?

(Probe) how were you able to choose acceptance (rather than resistance, avoidance or reacting)? What did the feeling feel like in your body? Tell me more…

**Construct questions**

1. Can you tell me about a time, at work or at home, that you noticed using one of the models we just talked about impacted your experiences (or feelings) of [self-compassion/burnout/imposter syndrome/moral injury]?

**Coaching Intervention Takeaways/Feedback**

1. How do you see this coaching program impacting your work as a doctor?

(Probe) How has this program impacted your experience of [self-compassion/burnout/imposter syndrome/moral injury] as a doctor so far?

(Probe) How do you see this program impacting your experience of [self-compassion/burnout/imposter syndrome/moral injury] in the future?

1. How do you see this coaching program impacting your overall life (in and outside of work)?

(Probe) How does this impact your self-compassion?

1. Can you describe to me your biggest "ah-ha" moment in this program? When did you have this moment, and why did this particular thought stick with you?

(Probe) Can you give me an example of a time you have or will apply to this to your work?

1. Which modality or combination of modalities resonated with you the most for coaching?

(Probe) Live coaching calls, website, worksheets, ask-for-coaching written coaching, YouTube webinars?

(Probe) Why that method?

(Probe) Why not other methods?

1. Did you attend any live coaching calls? If so, about how many (ballpark)?

(Probe) tell me about attending the calls as a listener?

(Probe) what did you like or not like about watching your colleagues get coached?

(Probe) are there any other effects from watching the calls?

1. If you attended any live coaching calls - did you get coached live? If so, how many times (ballpark)?

(Probe) tell me about getting coached live - what was that like for you?

(Probe) what did you like or not like about getting coached?

(Probe) how did you feel about the group-coaching format?

1. Did you ever watch recorded coaching calls? How many? How was that for you?
2. Did you use the Ask-for-coaching function? If so, how many times?

(Probe) what did you like or not like about this anonymous written coaching

1. Did you ever read others ask-for-coaching submissions and responses? If so, did you get anything out of it? Tell me more…
2. Did you watch any of the webinars (online 3–5-minute videos on the website)? Tell me more…
3. Did you do any of the worksheets? Tell me more…
4. Are there any recommendations you would like to provide to enhance the program in the future?

(Probe) Adapt the format, further content to cover?

(Probe) Imagine you were designing this program, are there other ways in which we could build a sense of community?

**Wrap Up**

1. Reflecting back on your expectations for the program, can you describe how the Better Together program met your needs and expectations? Can you describe any ways that the program did not meet your needs or expectations?

1. After engaging in this interview, are there any particular comments or takeaways you would like to ensure are emphasized in the summary of the findings?

1. After the research study ends in December, how would you like to continue to engage in coaching?

1. When we have findings from this, would you like to see them?

*Thank you for this information. As a next step, the recording of this interview will be transcribed. After interviews have been completed, we will deliver interview transcripts to our research assistants and Dr. Mann and Dr. Fainstad for their review. This should occur in approximately July and August of 2021. We very much appreciate your willingness to participate in these interviews. The Better Together Coaching Program is grateful for your responses and appreciative of your time.*

*If you have any follow—up questions or concerns, you can reach me at* [*pari.shah@du.edu*](mailto:pari.shah@du.edu)

**Appendix C.**

*Themes & Participant Quotes Table- a selection of quotes from 12 of 17 participants in the qualitative study of Better Together Physician Coaching pilot. University of Colorado, 2021.*

| **Appendix D. Themes & Participant Quotes** | | |
| --- | --- | --- |
| Theme | Subtheme | Quote |
| Using Metacognition as a Tool for Healthy Coping | Countering Burnout | *“I think stepping back with the coaches and realizing that the circumstance was just that I'm a doctor or I work in the ICU, and that everything else, all of that anxiety and worry and torturing myself, is stuff that I created with my thoughts” (Participant 1, surgical field).* |
|  |  | *“Wow, I really do resonate with some of these questions that they're asking about not feeling good at work and feeling frustrated with my patients and feeling these feelings that I've never felt before. I was like, I really do feel burnt out” (Participant 2, surgical field).* |
|  | Growing Self-Compassion | “I got something out of the program, [and] it was to be a lot more gentle with myself” *(Participant 3, nonsurgical field).* |
|  |  | *“I think just that grace that's granted when you ask yourself like, "What would be enough?"  I think just hearing that phrase, what would be enough for you to say that you've done enough for today or to have met this goal and realizing that it didn't have to be 100% but to still be acceptable to both myself” (Participant 4, nonsurgical field).* |
|  |  | *“I think having that grace towards yourself definitely can make you more confident as a physician, I think more happy as a physician. I definitely love what I do, but that doesn't mean there's not challenges. So, I think having these frameworks helps with being more equipped when there's something tough that comes up” (Participant 4, nonsurgical field).* |
|  | Managing Imposter Syndrome and Perfectionism | *“[Because of the program] I think I just allowed myself to be imperfect sometimes, and now I feel like I don't even like saying ‘imperfect’. I don't like focusing on the things I may have thought as bad before at all. I think I've really shifted my mindset to say anything that maybe had a poor outcome or wasn't what I wanted is all just a growth period for me”* *(Participant 5, nonsurgical field).* |
|  |  | *“[Prior to BT] I thought that constructive feedback meant that I was a bad doctor, and that I was bad at operating, whereas she was literally just helping me. And it was funny that I finally realized that my thoughts were, oh, I should be perfect at everything. I should know everything and no one should ever give me any feedback, like this unrealistic expectation that I didn't even realize.”* *(Participant 6, surgical field).* |
|  | Improving Relationships | *“I had two male seniors who would make jokes or make comments or demand more of me. And I felt like I was working really hard but still like not being respected... so we worked through a different model, for a different result...and I wrote in my planner, like their thoughts about you are their models and what they have going on and their thoughts about you are actually no reflection of how you are as a doctor or a teammate, or any of that. So really being able to separate these other people from my life and not letting them have as much control over my thoughts or any control over my thoughts was so helpful”* *(Participant 6, surgical field).* |
|  |  | *“It helped with my overall life in regard to my relationship with my boyfriend and my relationships with my parents”* (*Participant 7, nonsurgical field).* |
| Building Community | Sense of Community | *“I think connection to people, feeling like a sense of community, or that we all share something that we're going through. Learning based on their experiences” (Participant 8, surgical field).* |
|  |  | *“Other people can understand me. [They] were able to vocalize a lot of things that I was thinking” (Participant 9, nonsurgical field).* |
|  |  | “*I don't know if the program would have been different if it wasn't for COVID. Instead of over Zoom, these groups would be in person. Half of these things, that would have been a good idea because it's nice to have just feeling human interaction and bonding. And then half I'm like, the nice thing about the Zoom was that when you went up there, you didn't see anyone else looking at you. You kind of felt you were privately talking to someone”* (*Participant 9, nonsurgical field).* |
|  | Shared Experiences | *“I think a huge strength is just being able to see, ‘Oh my experience, although some things in my experience are unique, a lot of it isn't’. A lot of it is something that we all share of being residents, working really hard, feeling inadequate at times, wanting to be perfect, feeling like we are going to get found out. Those things, it was helpful to look around and be like, ‘Oh, there's all these amazing women who are awesome doctors who also feel all the same things in this…’” (Participant 8, surgical field).* |
|  |  | *“She was working on healthy eating and what her triggers were, and that was really helpful for me because I know that stress is very much a trigger for me. So that was helpful to watch her be really vulnerable about kind of a pretty vulnerable topic too”* (*Participant 3, nonsurgical field).* |
| Customizable Experience | Multimodal | *“I think allowing multiple ways to interact with the program. So not only the live coaching, but the ask-for-coaching and then being able to watch the videos on your own time, and then some people participated by just watching others. I was someone who was pretty regularly being coached live, which I found nerve wracking at first, but then probably the most beneficial part”* (*Participant 10, nonsurgical field)* |
|  |  | *“So, it's nice being able to have a format that you can ask it at any time and then also having a written response so that you can go back to it if you need to”* (*Participant 8, surgical field).* |
|  | Program Attributes | *“I thought they were very brief and to the point, so I could break up stuff into small chunks and I would learn about it. And then I can take some time to either reflect about what they were talking about or just go right to the worksheet”* (*Participant 11, nonsurgical field).* |
|  |  | *“Access and availability...if I were in an inpatient rotation where I just was not free by the time it started, I could go back and listen to the sessions later. I read through some of people's coaching requests. I was like, ‘Oh, yeah. This is useful for me, too’ in addition to other curriculum that you can go at your own pace for”* (*Participant 4, nonsurgical field 1).* |
|  |  | *“I think that it was nice that you could pick and choose from the level of commitment that you wanted”* (*P86, Internal Medicine, PGY 2).* |
|  |  | *“So, I really liked that they had an anonymous ask for coaching thing, or an ability to set up just a one-time meeting with them on their own. I thought that was great”* (*Participant 8, surgical field).* |
|  |  | *“I felt like maybe that wasn't a good fit because a lot of my co-residents were around. But I still feel like I benefited from watching other people get coached as well”* (*Participant 12, surgical field).* |
