# Supplementary material for: “We’re all going through it”: impact of an online group coaching program for medical trainees: a qualitative analysis
Source: BMC Med Educ. 2022 Sep 13;22:675. doi: 10.1186/s12909-022-03729-5 (PMC9468533; doi:10.1186/s12909-022-03729-5)
Supplement: Supplementary file 3 — Additional file 3. Consolidated criteria for reporting qualitative studies (COREQ): 32-item checklist. [file 12909_2022_3729_MOESM3_ESM.docx]

Consolidated criteria for reporting qualitative studies (COREQ): 32-item checklist

| No | Item | Guide questions/description | Reported on pg No |
| --- | --- | --- | --- |
| Domain 1: Research team and reflexivity |  |  |  |
| Personal Characteristics |  |  |  |
| 1. | Interviewer/facilitator | Which author/s conducted the interview or focus group?  *Pari Shah and Nathalie Dieujuste* | 7 |
| 2. | Credentials | What were the researcher's credentials? *E.g. PhD, MD*  *PS: LCSW, MSW, PhD Candidate*  *ND: MS, PhD Candidate* | 1 |
| 3. | Occupation | What was their occupation at the time of the study?  *PS: Social Worker, graduate student in the social work program at the University of Denver.*  *ND: graduate student in the Clinical Psychology PhD program at the University of Denver.* | 1 |
| 4. | Gender | Was the researcher male or female?  *PS: female*  *ND: female* |  |
| 5. | Experience and training | What experience or training did the researcher have?  *PS: Prior experience with qualitative and mixed methods research. Clinical experience as a LCSW.*  *ND: Prior experience with qualitative and mixed methods research. Project manager and research coordinator with the Rocky Mountain Regional VA Medical Center and the University of Colorado Anschutz Medical Campus. Her research experience spans several topic areas, including trauma, suicidology, health services research, and Black maternal health.* | 7 |
| Relationship with participants |  |  |  |
| 6. | Relationship established | Was a relationship established prior to study commencement?  *Yes* |  |
| 7. | Participant knowledge of the interviewer | What did the participants know about the researcher? e*.g. personal goals, reasons for doing the research* |  |
| 8. | Interviewer characteristics | What characteristics were reported about the interviewer/facilitator?  *Brief intro with reasons and interests in the research topic* |  |
| Domain 2: study design |  |  |  |
| Theoretical framework |  |  |  |
| 9. | Methodological orientation and Theory | What methodological orientation was stated to underpin the study?  *Constructivist grounded theory* | 8 |
| Participant selection |  |  |  |
| 10. | Sampling | How were participants selected?  *Purposive convenience sampling* | 7 |
| 11. | Method of approach | How were participants approached? E  *Email* | 7 |
| 12. | Sample size | How many participants were in the study?  *17* | 10 |
| 13. | Non-participation | How many people refused to participate or dropped out? Reasons?  *5 – unable to find a suitable time for the interview (22 responded to initial email, 17 were interviewed and included in the study)* | 10 |
| Setting |  |  |  |
| 14. | Setting of data collection | Where was the data collected?  *Workplace or home, interviews conducted over video conferencing* | 8 |
| 15. | Presence of non-participants | Was anyone else present besides the participants and researchers?  *No* |  |
| 16. | Description of sample | What are the important characteristics of the sample?  *See table 1: The participants ranged in age from 25 to 32, with an average post-graduate year of 2.35; the participants were majority white, and all were cis-female, and heterosexual. Seven different specialties were represented: otolaryngology, family medicine, general surgery, internal medicine, neurology, obstetrics and gynecology, and psychiatry.* | 10 |
| Data collection |  |  |  |
| 17. | Interview guide | Were questions, prompts, guides provided by the authors? Was it pilot tested?  *See Appendix B – yes it was pilot tested.* | Appendix B |
| 18. | Repeat interviews | Were repeat interviews carried out? If yes, how many?  *No* | n/a |
| 19. | Audio/visual recording | Did the research use audio or visual recording to collect the data?  *Audio* | 8 |
| 20. | Field notes | Were field notes made during and/or after the interview or focus group?  *Yes, during and after* | 8 |
| 21. | Duration | What was the duration of the interviews or focus group?  *Range: 26 to 70 minutes, with the majority lasting for one hour.* | 8 |
| 22. | Data saturation | Was data saturation discussed?  *Yes* | 8-9 |
| 23. | Transcripts returned | Were transcripts returned to participants for comment and/or correction?  *No, but the themes and findings were for a member check* | n/a |
| Domain 3: analysis and findings |  |  |  |
| Data analysis |  |  |  |
| 24. | Number of data coders | How many data coders coded the data?  *2* | 7 |
| 25. | Description of the coding tree | Did authors provide a description of the coding tree?  *yes* |  |
| 26. | Derivation of themes | Were themes identified in advance or derived from the data?  *Derived from data* | 8 |
| 27. | Software | What software, if applicable, was used to manage the data?  *Rev, RedCap* | 8 |
| 28. | Participant checking | Did participants provide feedback on the findings?  *Yes, a member check was done* | 9 |
| Reporting |  |  |  |
| 29. | Quotations presented | Were participant quotations presented to illustrate the themes / findings? Was each quotation identified?  *yes* | 11-18 |
| 30. | Data and findings consistent | Was there consistency between the data presented and the findings?  *yes* | 11-18 |
| 31. | Clarity of major themes | Were major themes clearly presented in the findings?  *yes* | 11-18 |
| 32. | Clarity of minor themes | Is there a description of diverse cases or discussion of minor themes?  *yes* | 11-18 |

*Developed from: Tong A, Sainsbury P, Craig J. Consolidated criteria for reporting qualitative research (COREQ): a 32-item checklist for interviews and focus groups. International Journal for Quality in Health Care. 2007. Volume 19, Number 6: pp. 349 – 357*
